# Ultraviolet B Photosensitivity in Rothmund–Thomson Syndrome Defined by Monochromator Phototesting

**DOI:** 10.1111/phpp.70129

**Published:** 2026-11-03

**Authors:** John Warner‐Levy, Donna Parkin, Jean Ayer

**Affiliations:** ^1^ The Faculty of Biology, Medicine and Health The University of Manchester Manchester UK; ^2^ Department of Dermatology, Cutaneous Biology Research Center Massachusetts General Hospital and Harvard Medical School Boston Massachusetts USA; ^3^ Photobiology Unit, Dermatology Centre, Salford Royal Hospital Northern Care Alliance NHS Foundation Trust, Manchester Academic Health Science Centre Greater Manchester UK

**Keywords:** action spectrum, minimal erythema dose, monochromator phototesting, *RECQL4*, Rothmund–Thomson syndrome, ultraviolet B photosensitivity

## 1 Introduction

Rothmund–Thomson syndrome (RTS) is a rare genodermatosis characterised by early‐onset poikiloderma, sparse hair, skeletal and dental abnormalities, and an increased risk of malignancy [1]. Photosensitivity is frequently reported, particularly in infancy and early childhood. However, objective abnormalities on formal phototesting are rarely documented. Existing reviews describe this photosensitivity as ill‐defined and distinct from the pronounced ultraviolet hypersensitivity observed in DNA repair disorders, such as Xeroderma Pigmentosum [1, 2]. Consequently, RTS is not generally classified among the classical photodermatoses.

To date, only a single case has demonstrated objective photosensitivity, reporting reduced minimal erythema doses (MEDs) to UVA with normal UVB responses [2]. We report the first case of RTS demonstrating a reproducibly reduced UVB MED on monochromator phototesting.

## 2 Case

A 15‐year‐old female of Ugandan descent with Fitzpatrick skin phototype V presented to photobiology with photosensitivity since 3 months of age. She had clinically diagnosed RTS from age 7, with supportive *RECQL4* sequencing findings comprising a pathogenic frameshift variant, c.1852del p.(Arg618fs), and a missense variant of uncertain significance, c.2360T>G p.(Leu787Arg) [3]. She was under the care of paediatric dermatology and oncology services and her current medications included regular emollient therapy, intermittent topical hydrocortisone/miconazole cream and vitamin D supplementation.

Approximately, 15 min of sun exposure elicited a painless, flat erythematous eruption that evolved into dry, scaly skin, with repeated exposures leading to fissuring. Episodes resolved over more than one week and were partially responsive to sunscreen.

Examination revealed extensive poikiloderma involving the face, neck, upper back and V‐area of the chest, with scleral melanosis on ocular examination. The dorsal hands demonstrated marked hyperkeratosis and lichenification over the interphalangeal joints, with squamous papillomas and palmar erythema and thickening. Hair density was preserved with focal thinning. Eyebrows and eyelashes were unaffected, and mild dental crowding was present. Representative clinical photographs are shown in Figure 1.

**FIGURE 1:**
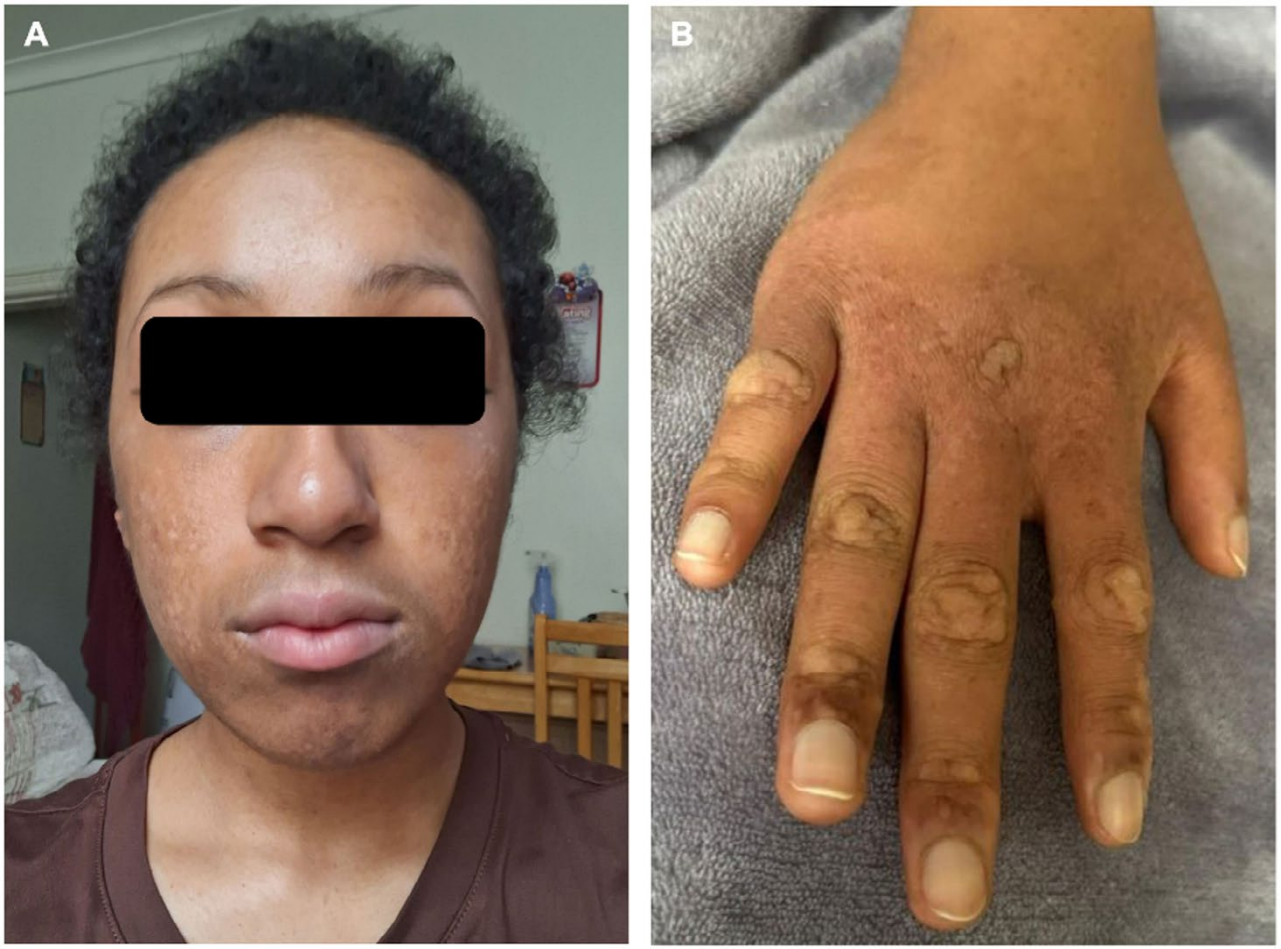
Facial poikiloderma (A) and dorsal hand involvement (B), including marked hyperkeratosis and lichenification over the interphalangeal joints, with squamous papillomas.

Laboratory evaluation demonstrated vitamin D insufficiency despite supplementation. Connective tissue disease screening and plasma porphyrin studies were negative, and serum IgE was within the reference range. The patient was prescribed broad‐spectrum SPF 50 sunscreen and cholecalciferol 800 IU daily, along with betamethasone valerate cream for facial flares, betamethasone dipropionate/salicylic acid ointment for hyperkeratotic lesions and emollient therapy for xerosis.

Monochromator phototesting demonstrated a reduced MED at 300 ± 5 nm (5 mJ/cm^2^), with normal responses across ultraviolet A and visible wavelengths. Provocation responses were assessed immediately and at 24 h. Broadband UVA provocation was performed on the right arm at 15 J/cm^2^. The first exposure produced no immediate response and mild erythema at 24 h. Subsequent exposures produced mild erythema and tanning immediately, with faint erythema and tanning or tanning alone at 24 h. Solar simulator provocation was performed on the left arm at 10 J/cm^2^. The first exposure was negative at both readings. Subsequent exposures produced faint‐to‐mild erythema immediately, with mild erythema and micropapules at 24 h after the third exposure. Patch and photopatch testing to a broad range of sunscreens and NSAIDs were negative. Detailed phototesting data are provided in Data S1.

In view of the marked photosensitivity and the presence of hyperkeratotic lesions over the interphalangeal joints, the case was reviewed at a national photodermatology multidisciplinary meeting. Further rheumatological evaluation was undertaken to exclude a coexisting connective tissue disease as an alternative or contributing cause of photosensitivity. Antinuclear antibodies were positive at a titre of 1:2560, while anti‐double‐stranded DNA antibodies, extractable nuclear antigen antibodies, and anticardiolipin antibodies were negative, and lupus anticoagulant was not detected. The high antinuclear antibody titre raised consideration of a lupus‐spectrum connective tissue disease, although negative disease‐specific serology made this less likely. Complement levels, immunoglobulins and erythrocyte sedimentation rate were normal. Myositis‐specific antibodies, cryoglobulins and viral screening were negative. Repeat monochromator phototesting performed 10 months after the initial study reproduced the action spectrum, with a persistently reduced MED at 300 nm (3.5 mJ/cm^2^).

Histological examination of a biopsy obtained from an erythematous lesion on the right upper back demonstrated a mild spongiotic pattern with a superficial perivascular lymphocytic infiltrate, without basement membrane thickening or increased dermal mucin (Figure 2). Direct immunofluorescence of non‐lesional skin revealed isolated linear IgM deposition at the basement membrane zone, with otherwise negative staining. Although the high antinuclear antibody titre and isolated linear IgM deposition raised consideration of a lupus‐spectrum connective tissue disease, the absence of disease‐specific serological markers, normal complement levels and inflammatory markers, non‐diagnostic histology and specialist rheumatology assessment argued against this diagnosis.

**FIGURE 2:**
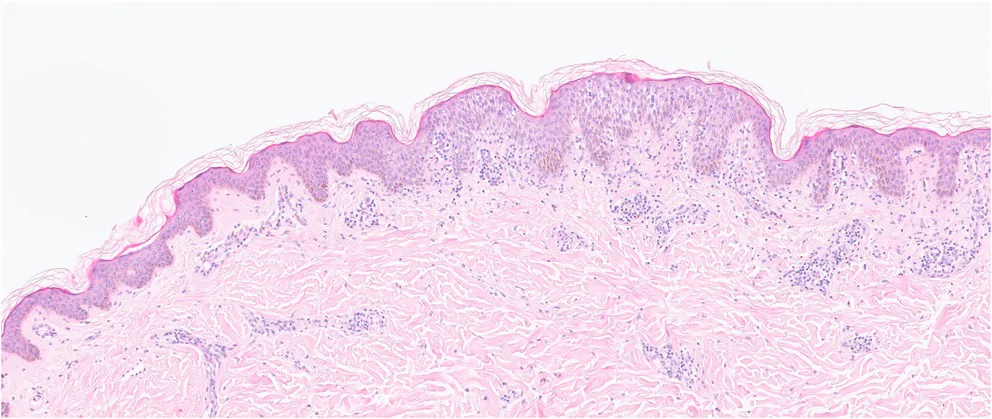
Lesional biopsy showing mild spongiosis and superficial perivascular lymphocytic inflammation.

## 3 Discussion

RTS is genetically heterogeneous, with molecular subtypes described as *ANAPC1*‐associated RTS1, *RECQL4*‐associated RTS2, *CRIPT*‐associated RTS3 and *DNA2*‐associated RTS4 [4]. In the present case, RTS had been diagnosed clinically in childhood and was accompanied by supportive *RECQL4* sequencing findings, making RTS2 the most likely subtype. However, definitive molecular confirmation was limited by the classification of one *RECQL4* missense variant as a variant of uncertain significance and the absence of parental phasing.

This case demonstrates objective and reproducible reduction in the MED at 300 nm in a patient with clinically diagnosed RTS and supportive *RECQL4* findings, extending the reported photobiologic spectrum beyond the previously described case of UVA sensitivity. The reproducibly reduced 300 nm threshold supports a genuine photobiologic abnormality rather than subjective sunlight intolerance, particularly notable in Fitzpatrick skin phototype V, in which higher ultraviolet thresholds would be expected.

The observed action spectrum, with a reduced MED at 300 nm but normal responses at UVA and visible light wavelengths, is consistent with the wavelength dependence of UV‐induced DNA photoproduct formation [5]. This pattern suggests that photosensitivity in RTS may reflect impaired tolerance to ultraviolet‐induced DNA damage, which is biologically plausible given the role of *RECQL4* in DNA replication and repair [6, 7]. Whether this represents an under‐recognised manifestation of RTS, phenotypic heterogeneity or coincident photosensitivity requires further study.

## Funding

The authors have nothing to report.

## Consent

Written informed consent was obtained for publication of this case and images.

## Conflicts of Interest

The authors declare no conflicts of interest.

## Supporting information


**Data S1:** Phototesting findings: wavelength‐specific MEDs and provocation testing.

## Data Availability

The data that supports the findings of this study are available in the Supporting Information of this article.
