# Supplementary material for: Ultraviolet B Photosensitivity in Rothmund–Thomson Syndrome Defined by Monochromator Phototesting
Source: Photodermatol Photoimmunol Photomed. 2026 Nov 3;42(6):e70129. doi: 10.1111/phpp.70129 (PMC13731749; doi:10.1111/phpp.70129)
Supplement: Supplementary file 1 [file PHPP-42-e70129-s001.docx]

**Phototesting findings: wavelength-specific MEDs and provocation testing**

**September 2024**

Monochromator phototesting demonstrated a reduced minimal erythema dose (MED) at 300 ± 5 nm (5 mJ/cm²). Responses at 320 ± 5 nm (2.5 J/cm²), 330 ± 5 nm (7 J/cm²), 350 ± 5 nm (40 J/cm²), 370 ± 7.5 nm (28 J/cm²), and 400 ± 7.5 nm (113 J/cm²) were within normal limits. No erythemal response was elicited during visible-light testing at 500 ± 12.5 nm or 600 ± 12.5 nm.

Broadband UVA provocation testing was performed on the right arm using repeated 15 J/cm² exposures. Following the first exposure, there was no immediate reaction, with mild erythema at 24 hours. Following the second exposure, corresponding to a cumulative dose of 30 J/cm², mild erythema and tanning were observed immediately, with faint erythema and tanning at 24 hours. Following the third exposure, corresponding to a cumulative dose of 45 J/cm², tanning was observed at 24 hours.

Solar simulator provocation testing was performed on the left arm using repeated 10 J/cm² exposures. Following the first exposure, there was no immediate or 24-hour reaction. Following the second exposure, corresponding to a cumulative dose of 20 J/cm², faint erythema was observed immediately and at 24 hours. Following the third exposure, corresponding to a cumulative dose of 30 J/cm², mild erythema was observed immediately, with mild erythema and micropapules at 24 hours.

**May 2025, 10 months later**

Repeat monochromator phototesting demonstrated a persistently reduced MED at 300 ± 5 nm (3.5 mJ/cm²). The response at 320 ± 5 nm was within normal limits (1 J/cm²). No erythemal response was elicited at 330 ± 5 nm, 350 ± 5 nm, 370 ± 7.5 nm, or 400 ± 7.5 nm, or during visible-light testing at 500 ± 12.5 nm or 600 ± 12.5 nm.
